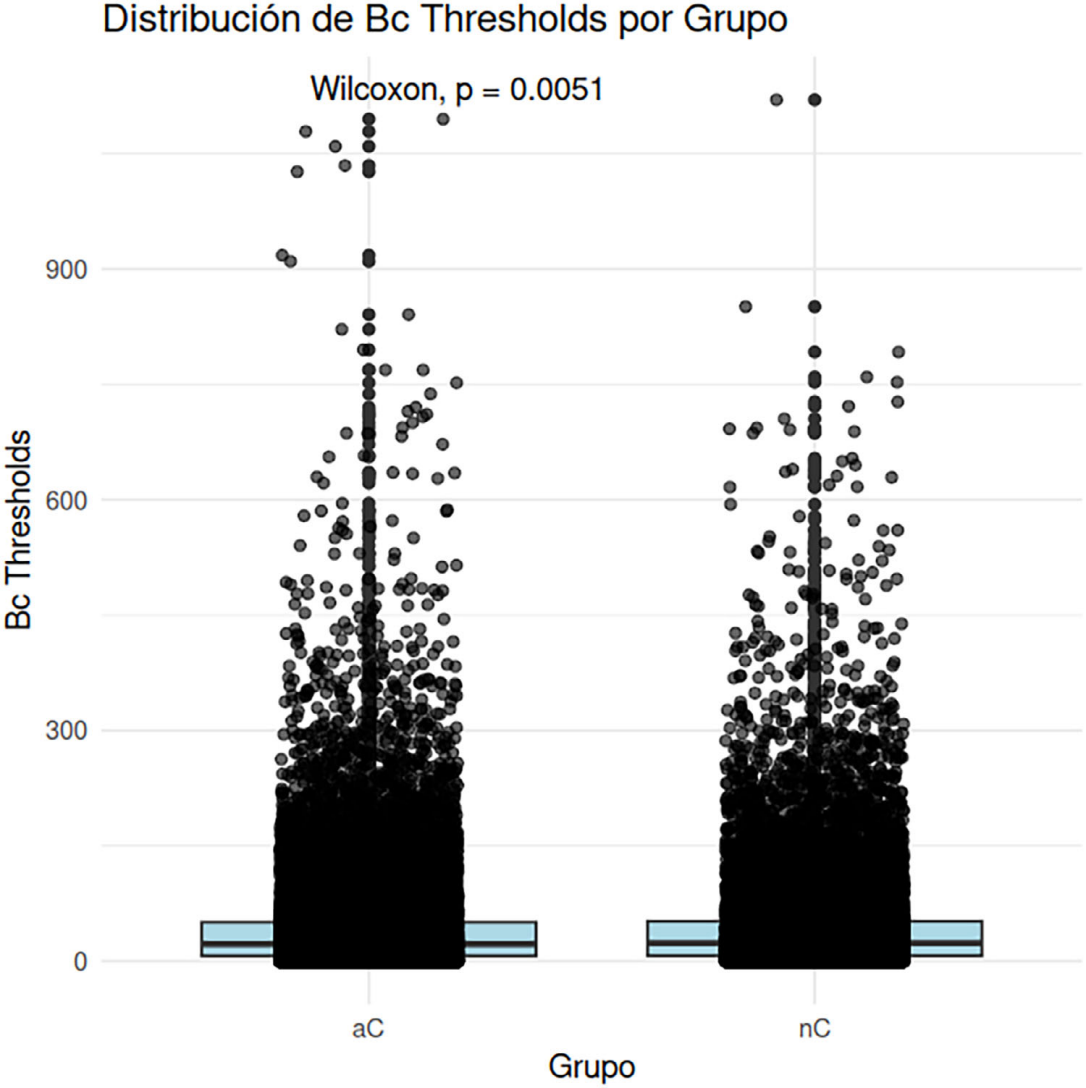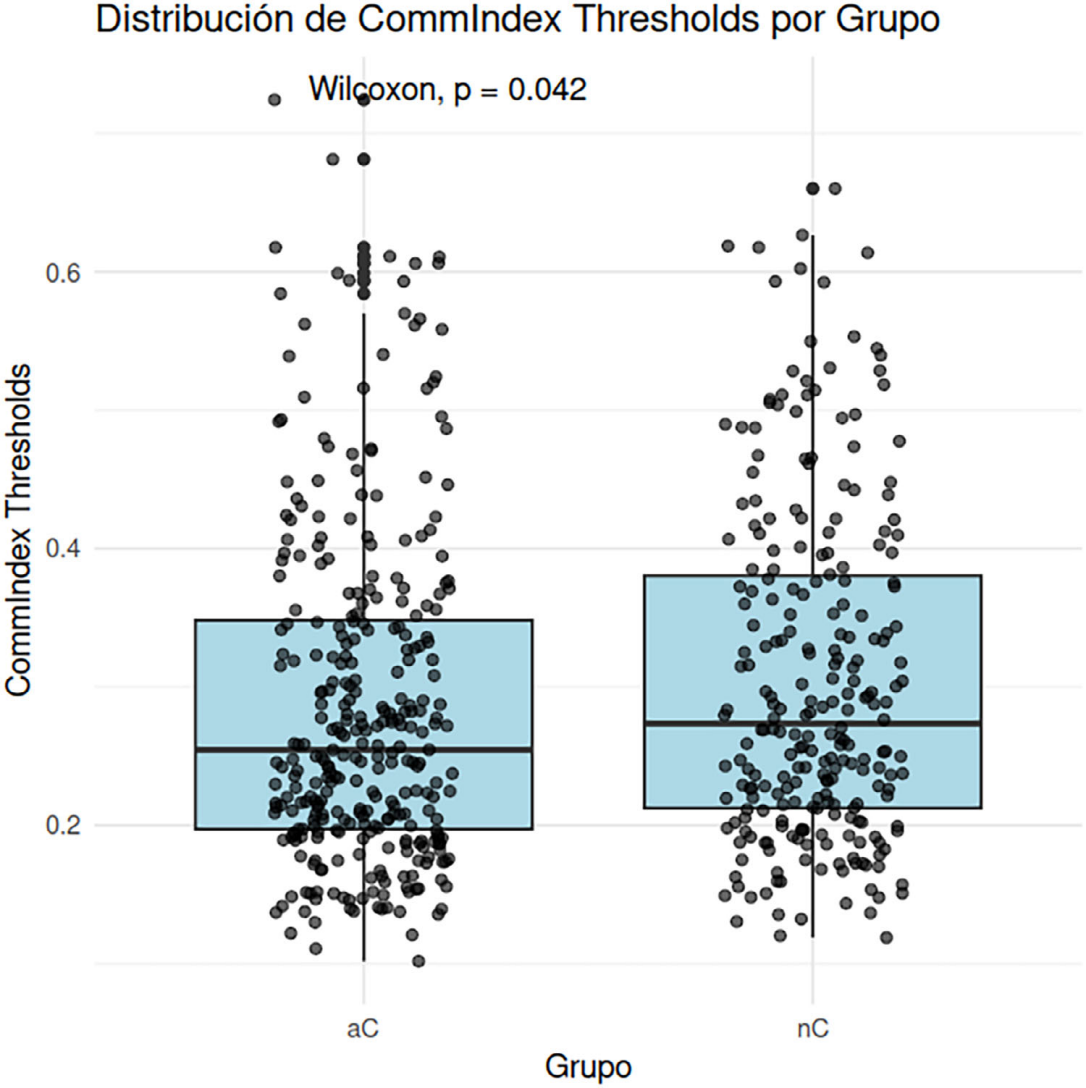# Betweenness centrality as neurophysiological marker of DMN functional connectivity in asymptomatic carriers of PSEN1 E280A for ADAD

**DOI:** 10.1002/alz70856_104007

**Published:** 2025-12-26

**Authors:** Claudia Aponte, Laura García, María José Hidalgo Ramírez, Lisset Zea, Carlos Andrés Tobón Quintero, John Fredy Ochoa Gómez, David Fernando Aguillón Niño

**Affiliations:** ^1^ Grupo Neuropsicología y Conducta, Universidad de Antioquia, Medellín, Antioquia, Colombia; ^2^ Grupo de Neurociencias de Antioquia, Facultad de Medicina, Universidad de Antioquia, Medellín, Antioquia, Colombia; ^3^ Grupo Neuropsicologia y Conducta (GRUNECO), Medellin, Antioquia, Colombia; ^4^ School of Engineering, University of Antioquia, Medellin, Antioquia, Colombia; ^5^ Grupo Neuropsicología y Conducta GRUNECO, Universidad de Antioquia, Medellín, Antioquia, Colombia; ^6^ Grupo de Neurociencias de Antioquia, Universidad de Antioquia, Medellin, Antioquia, Colombia; ^7^ Grupo Neuropsicologia y Conducta (GRUNECO), Universidad de Antioquia, Medellin, Antioquia, Colombia; ^8^ Grupo Neuropsicología y Conducta, Facultad de Medicina, Universidad de Antioquia, Medellin, Antioquia, Colombia; ^9^ School of Medicine, University of Antioquia, Medellín, Antioquia, Colombia; ^10^ Grupo Neuropsicología y Conducta (GRUNECO), Universidad de Antioquia, Medellín, Antioquia, Colombia; ^11^ Grupo de Neurociencias de Antioquia, Universidad de Antioquia, Medellín, Antioquia, Colombia; ^12^ School of Engineering, University of Antioquia, Medellín, Antioquia, Colombia

## Abstract

**Background:**

Graph metrics have been used to evaluate functional connectivity in Alzheimer's disease (AD). It has been found that nodal and global graph metrics are altered in subjects with AD and MCI compared to controls. In subjects with mutations associated with autosomal dominant Alzheimer's disease (ADAD), these metrics may vary depending on the stage within the disease continuum. This study aimed to evaluate the characteristics of graph metrics in the DMN specifically in asymptomatic subjects carrying the PSEN1 E280A mutation.

**Methods:**

32 asymptomatic carriers (aC) and 25 non‐carrier controls (nC) matched by sex, age and education status, underwent rs‐fMRI acquisition for 10 minutes. Pre‐processing of images was performed with CONN toolbox and RSN were extracted with Schaefer atlas with 79 ROI for DMN. Graph metrics were extracted with GRETNA toolbox, with a range of sparsity thresholds from 0.05‐0.5 for all metrics. A non parametric t‐test wilcoxon and linear mixed model (LMM) were performed, with corrections for multiple comparisons with Bonferroni.

**Results:**

Significant differences were found in education (*p*‐value < 0.001, confidence interval 95% 1.43 – 5.15), but not for age and sex. Betwenness Centrality (Bc) was higher in nC tan aC (*p*‐value 0.0051, Cohen's d estimate: ‐0.011 CI95% ‐0.030 to 0.007) and Modularity (Q) (*p*‐value 0.042, Cohen's d estimate: ‐0.15 CI95% ‐0.318 to 0.0136). Regarding the LMM, differences were observed in the intercepts of nodal graph metrics, but no differences were found between groups. However, when examining each intercept individually, it was shown that clustering coefficient, betweenness centrality, local efficiency, and degree centrality increase the slope in nC, while they decrease it in aC. At the global graph level, no differences were found between aC and nC groups.

**Conclusion:**

Bc idifferentiates between aC and nC. It is higher in nC, indicating that, at a local level, there is a greater number of nodes through which more connections pass, making them central to DMN connectivity. The local changes in aC are attributed to pathophysiological alterations in information distribution and the reorganization of DMN to distribute information across RSN and preserve cognitive functions.